# The -938C>A Polymorphism in* MYD88* Is Associated with Susceptibility to Tuberculosis: A Pilot Study

**DOI:** 10.1155/2016/4961086

**Published:** 2016-12-29

**Authors:** Kalliopi Aggelou, Elena Konstantina Siapati, Irini Gerogianni, Zoe Daniil, Konstantinos Gourgoulianis, Ioannis Ntanos, Emmanouel Simantirakis, Elias Zintzaras, Vassiliki Mollaki, George Vassilopoulos

**Affiliations:** ^1^Department of Respiratory Medicine, University of Thessaly Medical School, Larissa, Greece; ^2^9th Department for Chest Diseases, “Sotiria” Hospital, Athens, Greece; ^3^Cell and Gene Therapy Laboratory, Center for Basic Research II, Biomedical Research Foundation of the Academy of Athens, Athens, Greece; ^4^Department of Biomathematics, University of Thessaly School of Medicine, Larissa, Greece; ^5^Hellenic National Bioethics Committee, Athens, Greece; ^6^Department of Medical Laboratories, Faculty of Health and Caring Professions, Technological Educational Institution of Athens, Athens, Greece; ^7^Division of Haematology, University of Thessaly Medical School, Larissa, Greece

## Abstract

*Introduction*. Tuberculosis (TB) is a major disease worldwide, caused by Mycobacterium tuberculosis (MTB) infection. The Toll-Like Receptor (TLR) pathway plays a crucial role in the recognition of MTB.* Aim*. The present study aimed to investigate the involvement of myeloid differentiation primary response protein 88 (*MYD88*) gene polymorphisms in TB.* Materials and Methods*. A total of 103 TB cases and 92 control subjects were genotyped for the* MYD88 -*938C>A (rs4988453) and 1944C>G (rs4988457) polymorphisms.* Results*. The* MYD88* -938CA and -938AA genotypes were associated with an increased risk for tuberculosis with odds ratio (OR) of 5.71 (95% confidence intervals [CIs] 2.89–11.28, *p* = 0.01).* Conclusions*. The* MYD88* -938C>A genetic polymorphism is associated with increased susceptibility to TB and may serve as a marker to screen individuals who are at risk.

## 1. Introduction

Tuberculosis (TB) is a chronic, usually airborne, infectious disease caused mainly by* Mycobacterium tuberculosis, *Mtb. TB is responsible for approximately 1.5 million deaths per year [1]. After inhalation of infected aerosols into the host lungs, mycobacteria first encounter the alveolar macrophages and get phagocytosed. Mycobacteria that escape the initial intracellular destruction can multiply and disrupt the macrophage. This results in further chemokine release and recruitment of monocytes and other inflammatory cells to the site. However, Mtb inhibit phagosome maturation in macrophages and, along with lysosomal fusion, they can survive intracellularly [2]. Two to three weeks after infection, antigen-specific T lymphocytes are recruited at the early lesions (tubercles) and release proinflammatory cytokines such as interferon-*γ* (IFN-*γ*), in a second attempt to activate macrophages and kill the intracellular mycobacteria. This results in the formation of a granuloma which is a structure consisting of macrophages, epithelioid cells (differentiated macrophages), and multinucleated giant cells (also known as Langerhans giant cells), surrounded by T lymphocytes. The granuloma provides the Mtb with a long-term survival shelter where the microorganisms are protected from the immune response [3]. Finally, another defense mechanism for the control of intracellular pathogens is autophagy; in macrophages, activation of autophagy leads to phagosome maturation, increased acidification, and killing of mycobacteria [4].

Innate immunity, as mediated by Toll-Like Receptors (TLRs), plays a central role in TB response. TLRs recognize several components of* M. tuberculosis *and activate signaling pathways leading to an immune activation that is mediated by the myeloid differentiation primary response protein 88 (MyD88). MyD88 is significant for the protection against TB as evidenced by the fact that mice with a complete germline deletion of* MYD88* are particularly susceptible to infection [5–8]. These preclinical data indicate that MyD88 should, to a certain degree, be involved in the human immune response to TB. In fact, a number of human studies have confirmed these preclinical observations. MyD88 deficiency has been linked to susceptibility for invasive pneumococcal disease [9] and infections from* S. aureus* and* P. aeruginosa* [10]. In addition, deletion of* MYD88 *phenocopies* IRAK4 *deletion since both interrupt a pathway critical for pathogen sensing (TLR) and inflammation (IL-1R); patient's lymphocytes failed to mount immune responses in vitro to TIR agonists [11], recapitulating the disease phenotype and arguing for the central role of MYD88 in the immune response.

MyD88 plays a central role in the activation of the innate immune response to* M. tuberculosis*. Compared to wild-type mice,* MYD88* knockout mice are more susceptible to infection [2, 12]. Experimental data show exacerbated granulomatous inflammation and necrosis in the lungs of* MYD88*-/- mice relative to normal controls, a difference that gets more pronounced at 5 weeks after infection. It has also been found that IL-12, IFN-*γ*, and TNF, which are implicated in mycobacterial resistance in humans, are reduced significantly in the lungs of infected* MYD88*-deficient animals [13].

Granulomas generally form in order to confine pathogens, restrict inflammation, and protect surrounding tissue. Cytokines play a pivotal role in the development of granulomas while the significant task of activating the central immune response transcription factor NF*κ*-Β, is performed by MyD88. We have previously shown that certain* TLR9* and/or* MYD88* haplotypes are associated with diseases that are characterized by the presence of granulomas, such as Hodgkin's lymphoma [14] and sarcoidosis [15]. Taking into account that the development and accumulation of granulomas constitute the fundamental abnormality in TB, it is tempting to hypothesize that genetic polymorphisms in* MYD88*, the common TLR signaling molecule, are associated with the disease. The* MYD88* gene is located on chromosome 3p22 and consists of five exons [16]. The Single Nucleotide Polymorphisms (SNPs) -938C>A (dbSNP rs4988453) and 1944C>G (dbSNP rs4988457) define the two most common haplotypes in Caucasians [17]. Thus, the present study aimed to investigate the association of MYD88 genetic polymorphisms with tuberculosis in a Caucasian population.

## 2. Materials and Methods

### 2.1. Subjects

Peripheral blood samples were obtained from 103 TB patients and 92 control subjects of Caucasian origin from the University Hospital of Larissa (Larissa, Greece). Patients were diagnosed with TB by culture positive or smear-positive microscopy and satisfied the World Health Organization criteria for tuberculosis. Control subjects had a negative history for TB or any other disease. Informed consent was obtained from all patients and control subjects, and the protocol was approved by the Larissa University Hospital Ethics Committee.

### 2.2. Genotyping

Case and control subjects were genotyped for the* MYD88* (GenBank Accession Number NM_002468) SNPs -938C>A and 1944C>G, as previously described [14]. Briefly, the MyD88 938C>A genotypes were determined by PCR amplification of a 503 bp fragment of the 5′ flanking region of MyD88 using primers 5′ GCA GCC AGG ACC GCT TACT GC T 3′ (forward) and 5′ GCA CGT GGC CTT GCC CTT GCC CTT TAG G 3′ (reverse). The product was digested by* Bsr*I and the expected fragment sizes were as follows: 23, 97, 165, and 218 bp in the presence of the C allele and 97, 165, and 241 bp in the presence of the A allele.

### 2.3. Statistical Analysis

The chi-square test was used to test the association between genotype distribution and clinical status. The codominant and additive models were considered since they are orthogonal [18–20]. The magnitude of associations was expressed in terms of odds ratios (ORs) with the corresponding 95% confidence interval (CI). A multivariable analysis was not considered since the study was age and sex matched. The mode of inheritance was estimated using the degree of dominance index (*h*-index) [18, 19].

The association between cases and control subjects was also examined using OR_G_, which is a genetic model-free approach and provides an estimate of the overall risk effect by utilizing the complete genotype distribution. OR_G_ shows how many cases/control subjects pairs exist in the study for which the cases have larger mutational load relative to the number of pairs for which the healthy controls have the larger mutational load. Alternatively, OR_G_ indicates whether the mutational load of a variant is implicated in disease susceptibility [19, 21]; then, OR_G_ expresses the probability of a subject being sick relative to the probability of being healthy, given that diseased subjects have higher mutational load than the healthy subjects.

In healthy controls, deviation of the genotype distribution from the Hardy-Weinberg equilibrium (HWE) and existence of linkage disequilibrium (LD) between polymorphisms were evaluated using exact tests according to Weir [22, 23]. A result was considered statistically significant when *p* < 0.05. The ORs were estimated using SPSS (SPSS Inc., released 2003, Version 13, Chicago). HWE and LD were tested using the Genetic Data Analysis (GDA) software created by Lewis and Zaykin [24]. The haplotype frequencies were estimated and compared by SHEsis [25]. OR_G_ was calculated using ORGGASMA [21].

## 3. Results

### 3.1. Demographic Characteristics of the Study Population

A total of 103 TB cases and 92 controls were analyzed in this study. The mean age (±s.d.) was 42.9 ± 18.5 and 35.5 ± 10.1 years for cases and controls, respectively. There were 76 (73.8%) males and 27 (26.2%) females in the case group, whereas the control group comprised 27 (29.3%) males and 65 (70.7%) females. The control subjects were age- and sex-matched with the cases.

### 3.2. Genotype Distributions

Table 1 shows the genotype distributions for the two* MYD88* SNPs in cases and control subjects and the respective ORGs. Control subjects were conformed to HWE for both variants (*p* ≥ 0.05). Significant association between disease and genotype distribution was shown for* MYD88* -938C>A (*p* < 0.01).

Subsequently, OR_G_ produced significant results for the variant* MYD88* -938C>A [ORG = 5.71 (2.89–11.28)], indicating that the risk of disease is related to the mutational load of the variants. In particular, for any two subjects (TB and healthy), the probability of being diseased is almost six times higher (relative to the probability of being nondiseased) given that the diseased subject has higher mutational load than the healthy one. Alternatively, a subject has almost six times higher risk of disease relative to the risk of being healthy given that the subject with disease has a higher mutational load than the healthy subject.

Since significant association was shown for the* MYD88* -938C>A SNP, the additive and codominant models were tested (Table 2). A nonsignificant association for the additive model (*p* = 0.14) was observed, whereas a significant association (*p* < 0.01) was shown for the codominant additive model [OR = 5.58 (2.80–11.12)]. We then tested the mode of inheritance for the mutant allele and found that the mutant allele -938A behaves as a dominant allele for the risk of disease (*h* = 0.79), indicating that the homozygous -938AA has a greater risk of being sick from TB compared to the homozygous -938CC, and that the heterozygous -938CA has a risk of disease closer to the -938AA homozygous than to the midpoint between the two homozygotes (Table 3). Both SNPs were found to be in linkage disequilibrium (LD) (*p* < 0.05). D primes for LD testing between -938C>A and 1944C>G were 0.80 and 0.59 in cases and controls, respectively.

Table 3 presents the distribution of the estimated haplotype frequencies for the two SNPs (SNP1:* MYD88* -938C>A, SNP2:* MYD88* 1944C>G) in patients and control subjects, with the overall difference being significant (*p* < 0.01). Investigating the individual haplotypes (SNP1-SNP2), A-C, C-C, and C-G also derived significant results (*p* < 0.01). Haplotype A-C contributes to the risk of disease whereas haplotypes CC and CG may confer protection.

## 4. Discussion

The present study investigated whether genetic polymorphisms in* MYD88* are associated with TB. According to our results, the* MYD88* -938A allele is associated with an approximate 5.5-fold increased risk of TB, whereas no association was found for the* MYD88* 1944C>G SNP. The genotype distributions of the SNPs examined were all in HW in the control group, indicating no population stratification. Haplotype analysis also showed that the* MYD88* -938A- 1944C haplotype conferred an increased risk of disease.

In any case, statistical limitations should be taken into account when interpreting the present findings. The present study constitutes a pilot study, with a small sample size resulting in relatively large ORs. Candidate-gene association studies have the tendency to lack the power to detect a statistically significant association, mainly because of the extremely large population necessary for adequate statistical power. In that context, to achieve a power of 80% to identify a modest genetic effect (i.e., odds ratio of 1.2) of a polymorphism present in 10% of individuals, a sample size of 10,000 subjects or more may be required [21]. This size is hard to achieve even in large reference centers, depending on the disease. Future collaborative studies may allow the pooling of data, providing more power to detect significant associations. Furthermore, genome-wide association studies may be able to replicate the validity of the present findings.

There are numerous studies in the literature investigating the influence of* TLRs* genetic variation to TB susceptibility in humans (reviewed in [6]). Several SNPs in* TLRs* have been shown to have an effect in host immune response and TB through various mechanisms, including modifying TLR surface expression and altering interaction with other molecules such as MYD88, resulting in reduced NF-kB activation and altered protein folding and function [6].

Nonetheless, studies examining the association of* MYD88* genetic polymorphisms with TB are limited and, in fact, this is the first study investigating the effect of* MYD88* -938C>A on susceptibility to TB. Sánchez and colleagues examined the association of* MYD88* 1944C>G (rs4988457) with TB and, in agreement with our results, found no association with the disease [26]. Various other SNPs in* MYD88* have been investigated with regard to TB susceptibility. The SNPs rs6767684 and rs7744 were not associated with the disease [26, 27], whereas the SNP rs6853 was found to be associated with resistance to pulmonary tuberculosis [8].

The exact mechanism through which* MYD88* genetic variation may be associated with susceptibility to TB remains to be elucidated. Data so far show that the* MYD88* -938C>A (rs4988453) resides in the promoter region of the gene and has been shown to decrease promoter activity [28]. This provides a possible explanation, since decreased MyD88 expression levels may confer reduced NF-kB activation and, consequently, susceptibility to infection by pathogens and development of TB.

*MYD88* -938C>A has also been previously associated with sarcoidosis [15]. Indeed, MyD88 is critical for dendritic cell's potential to induce the differentiation of naive T cells into effector T cells producing IFN-*γ*, while reactivation of MyD88 signaling in CD11c or lysozyme M-expressing myeloid cells during* Mycobacterium bovis *infection is sufficient to restore systemic and local inflammatory cytokine production and to control pathogen burden. TNF-*α* and IFN-*γ* are particularly important in promoting the formation and function of the granulomas, whereas IL-10 is one of the main negative regulators of the response in both sarcoidosis and TB [7, 15, 29].

In conclusion, our pilot study suggests that the* MYD88* -938C>A genetic polymorphism confers susceptibility to TB.

## Figures and Tables

**Table 1:** Distribution of *MYD88* genotypes among patients and control subjects.

SNP	Genotype	Patients *n* (%)	Controls *n* (%)	*p *value for HWE^*∗*^	*p *value for association^#^	OR_G_ (95% CI)
-938C>A	CC	41 (44.1)	72 (82.8)	0.38	<0.01	5.71 (2.89–11.28)
	CA	50 (53.8)	15 (17.2)			
	AA	2 (2.2)	0 (0.0)			

1944C<G	CC	83 (81.4)	67 (75.3)	0.18	0.38	0.70 (0.36–1.39)
	CG	19 (18.6)	22 (24.7)			
	GG	0 (0.0)	0 (0.0)			

^*∗*^ *p* value for HWE in controls; ^#^ *p* value and OR_G_ for testing the association between genotype distribution of each SNP and disease.

**Table 2:** Association of the *MYD88* -938C>A with TB.

SNP	Genetic model	OR (95% CIs)	*h-*index	Mode of inheritance	*p* value
*MYD88* -938C>A	Additive	8.74 (0.41–186.4)	0.79	Dominance of mutant allele A to risk of disease	0.14
	Codominant	5.58 (2.80–11.12)			<0.01

Odds ratio (OR) and the corresponding 95% confidence intervals (CIs) for testing the association between *MYD88* -938C>A and TB for the additive and codominant models along with the* h*-index and the respective mode of inheritance for the significant allele are shown.

**Table 3:** Estimated haplotype frequencies for the two *MYD88* SNPs (SNP1: -938C>A, SNP2: 1944C<G).

Haplotype MyD88 -938C>A SNP1-SNP2	Estimated frequencies		*p *value	*p *value global
	Patients	Controls		
*A-C*	0.199	0.029	<0.01	<0.01
*A-G*	0.086	0.059	0.33	
*C-C*	0.699	0.841	<0.01	
*C-G*	0.011	0.065	0.01	

*p* values for comparing each haplotype between cases and healthy controls and the global *p* value for comparing the overall difference in haplotypes are shown.
